# Congenital Hypothyroidism 3-Year Follow-Up Project: Region 4 Midwest Genetics Collaborative Results

**DOI:** 10.3390/ijns4020018

**Published:** 2018-06-17

**Authors:** Kupper A. Wintergerst, Erica Eugster, Karen Andruszewski, Mary Kleyn, Nancy Vanderburg, Joe Sockalosky, Ram Menon, Sharon Linard, Suzanne Kingery, Susan R. Rose, Julie Moore, Gina Gembel, Lisa Gorman

**Affiliations:** 1 Department of Pediatrics, Endocrinology, University of Louisville, Louisville, KY 40202, USA; 2 Department of Pediatrics, Endocrinology, Indiana University, Indianapolis, IN 46202, USA; 3 Michigan Department of Community Health, Lansing, MI 48933, USA; 4 Minnesota Department of Health, St. Paul, MN 55155, USA; 5 Children’s Hospitals and Clinics of Minnesota, St. Paul, MN 55102, USA; 6 Department of Pediatrics, Endocrinology, University of Michigan, Ann Arbor, MI 48109, USA; 7 Ohio Department of Health Laboratory, Reynoldsburg, OH 43068, USA; 8 Department of Pediatrics, Endocrinology, College of Medicine, University of Cincinnati, Cincinnati, OH 45229, USA; 9 Region 4 Midwest Genetics Collaborative, Michigan Public Health Institute, Okemos, MI 48864, USA

**Keywords:** congenital hypothyroidism, thyroid, newborn, neonatal, screening

## Abstract

To identify the 3-year follow-up management and education patterns of primary care clinicians and pediatric endocrinologists for children diagnosed with congenital hypothyroidism (CH) through newborn screening programs, the Region 4 Midwest Genetics Collaborative, made up of seven regional states (Illinois, Indiana, Kentucky, Michigan, Minnesota, Ohio, Wisconsin), performed a survey study of parents and physicians caring for children identified with CH. The clinicians and parents of 409 children with CH regionally identified in 2007 were invited to participate in a voluntary survey. Responses relating to treatment, monitoring practices, educational resources, genetic counseling, and services provided/received were collected from 214 clinicians and 77 parents. In total, 99% had undergone a confirmatory test following positive newborn screening and 55% had imaging at diagnosis, but only 50% were identified as having the etiology identified. Thyroid withdrawal challenge testing was the choice method for re-evaluating thyroid function, but the approach varied. Clinician and parent responses to education and genetic counseling also differed. Clinicians report face-to-face education as the most common method, with less than 50% providing handouts to patients. Only 14% of patients were referred to a genetics counselor. Of parents reporting on their educational experience, 86% received face-to-face education from a pediatric endocrinologist and 4% received education from a genetic counselor. Only 65%, however, were satisfied with their education. These survey data suggest a lack of a standardized approach to diagnosis, follow-up, education, and genetic counseling. This collaborative effort provides insight into developing three-year follow-up, education and genetic counseling guidelines for children diagnosed with CH.

## 1. Introduction

Through the Newborn Screening (NBS) Program, approximately 1900 infants with congenital hypothyroidism (CH) are detected annually in the United States, with the birth prevalence varying based on state screening methods [1,2]. In order to prevent permanent cognitive and physical delays, infants with CH must be quickly and properly identified and treated. In addition, clinical follow-up of these children is critical to ensure that they receive appropriate monitoring and management. The American Academy of Pediatrics (AAP) and the European Society for Pediatric Endocrinology (ESPE) have published guidelines for the treatment of CH which recommend follow-up of CH cases until at least three years of age [3,4].

While the significant benefits to infants diagnosed by NBS are undeniable, little follow-up data are available assessing the long-term effectiveness of the screening program. In 2011, the Region 4 Midwest Genetics Collaborative formed the CH 3-year Follow-up Workgroup (CH Workgroup) as part of a regional initiative to develop standardized long-term follow-up guidelines. Funded by the Health Resources and Services Administration (HRSA), Region 4, which includes Illinois, Indiana, Kentucky, Michigan, Minnesota, Ohio, and Wisconsin, is charged with improving the health of children and their families by promoting the translation of genetic medicine into public health and health care services. Made up of pediatric endocrinologists, state laboratory personnel, public health follow-up specialists, and parents of children with CH, the CH Workgroup sought to identify management and education patterns of pediatric endocrinologists and primary care providers for children diagnosed with CH by state NBS within the region. In light of reports that some children with CH were discontinuing thyroid hormone replacement without appropriate medical advice and were being lost to follow-up, the CH Workgroup specifically sought to identify management practices for re-evaluating the diagnosis of CH at or before three years of age [5,6].

In 2014, we reported on the variety of logistical, regulatory, and legal challenges that the CH Workgroup had to successfully overcome to complete the study as a potential roadmap for future multi-state public health efforts [7]. This present manuscript provides the results from our initiative, and reviews 3-year follow-up recommendations to improve child health outcomes for children diagnosed with CH.

## 2. Materials and Methods

### 2.1. Subjects

The inclusion criteria for participation in the study were: diagnosis of CH through state NBS, and date of birth between 1 January 2007 and 31 December 2007. The NBS record contains data such as the child’s name, date of birth, mother’s name, maternal address and phone number provided at birth, and the clinician identified at birth as being responsible for the child’s care. After obtaining Institutional Review Board (IRB) approval (SR-2012-067, 4-Jan-2012), the state laboratory and/or health department accessed its own records to identify children diagnosed with CH who met the inclusion criteria. As this was a survey study, direct medical records, including any confirmatory testing results, were not reviewed, and, thus, the diagnosis of CH was purely based on NBS records and survey responses [7].

### 2.2. Study Design

The study was developed and executed under the guidance of Region 4 after IRB approval, and utilized the infrastructure provided by the HRSA grantee, the Michigan Public Health Institute (MPHI) [7]. The study was a basic survey collection with two mirrored surveys (one for clinicians and one for families) performed between 2012 and 2013.

The Clinician Survey instrument was adapted by the CH Workgroup. It includes questions relating to treatment and monitoring practices first used by the Michigan NBS Program to identify transient hypothyroidism at 3-year follow-up [6]. It also includes questions about educational resources, genetic counseling, and services provided to the family.

The Parent Survey was developed to identify parent preferences for educational resources and reasons children may have been lost to follow-up. It includes questions relating to treatment, educational resources, genetic counseling, and services they received, or would like to receive, from providers and the state’s health department, as well as questions pertaining to diagnosis and treatment.

Each respective state health department managed survey distribution, collection, de-identification, and submission to the MPHI for analysis. A cover letter with informed consent, the Clinician Survey, and a self-addressed stamped return envelope was mailed to the clinician identified through the procedure noted above. A similar, but abbreviated, method was used for the Parent Survey distribution. When the surveys were returned, the state health department assigned an identification (ID) code. The ID code was used in lieu of the subject’s name on all study data.

As described, de-identified data were sent from each state to the Region 4 headquarters at the MPHI for analysis. De-identified raw data were received from six of seven states. A record was entered for each unique ID code allowing for paired surveys from clinicians and parents to be matched within the same record. Once data collection was complete, analysis was completed by a centralized MPHI epidemiologist using IBM SPSS Statistics software (Version 22.0, Armonk, NY, USA:IBM Corp). A chi-square test for independence was used to test the relationship between parent satisfaction with information received from their clinician and the source of education.

## 3. Results

### 3.1. General

A total of 409 children were identified in Region 4 as having been diagnosed with CH in 2007 [7]. The combined birth prevalence was 1:1836 (Table 1). At the time of the survey, all 409 children identified with CH in 2007 were greater than 3 years of age. One state encountered barriers obtaining IRB approval through their NBS program due to public health record state law restrictions, and, therefore, no data were collected from this state for the study. In addition, one of the six states did not have access to parent names and addresses, so only clinician survey responses were collected. In total, the CH workgroup was able to aggregate parent data from five of the seven and clinician data for six of the seven states. A total of 334 Clinician Surveys and 291 Parent Surveys were able to be sent due to the above restrictions. The Clinician Survey response rate varied (21–100%) with an overall response rate of 64%. The Parent Survey response rate was 26%, with less variation among states (21–39%). We received paired Clinician and Parent Surveys for the same infant in 14% of surveys returned.

### 3.2. Diagnosis and Source of Care

Of the 214 clinician responses, 170 (79%) reported the patient on record was still actively being managed by their practice. Of the 44 clinicians who reported the patient on record was no longer being seen at their practice, 15 were lost to follow-up, 7 were listed as previous patients or referred to another doctor, 4 had never been seen at that practice, 3 had false positive test results or transient lab abnormalities, 3 were deceased, and the remaining 12 provided no reason.

**Figure 1:**
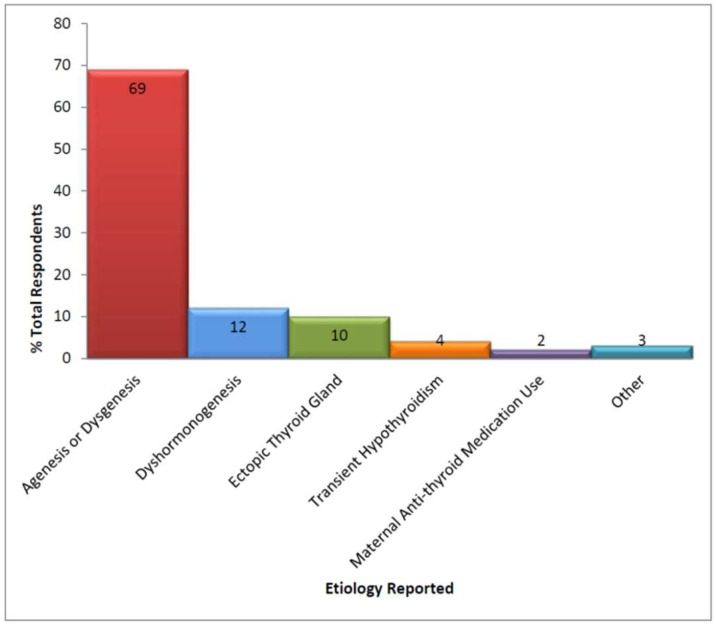
Etiology of congenital hypothyroidism *. *: No mutations in thyroid development genes, TSH receptors, defects in the hypothalamus/pituitary gland, or maternal iodine deficiency reported.

Of those CH patients continuing to be actively managed, 168 (99%) had at least one confirmatory serum blood test (median = 2.1, range from 1 to 4) with the most common being thyroid-stimulating hormone (TSH) and free thyroxine (T4). Thyroid ultrasounds and thyroid technetium scans were the most frequently used imaging modalities (Table 2), but only 15 patients were reported to have had both studies. The definitive etiology of the patient’s hypothyroidism was reported as having been established for only 50% of infants; 77 of 86 of these occurred within 6 months of initial diagnosis. Of those with a defined etiology (see Figure 1), clinicians reported agenesis/dysgenesis as the most common cause.

### 3.3. Thyroid Management and Re-Evaluation

#### 3.3.1. Clinician Responses

Of the clinician responses, 149 gave further details on thyroid management for patients described as still being on therapy, 26 were reported as being off therapy, and the remainder did not provide any further details. Clinicians reported that there were no plans to re-evaluate the diagnosis for 110 of the patients still being treated. Reasons provided included: the diagnosis was confirmed to clinician satisfaction at initial presentation (65), patient’s subsequent TSH levels since diagnosis required dosage changes (41), and patient was lost to follow-up (4). Of the 12 clinicians planning to re-evaluate, 7 planned to do so within the next six months, 1 within the next 6–12 months, and 4 within the next one to two years.

For the 26 patients identified as no longer receiving therapy, we only have further details for 19. Treatment was reported as having been stopped for 3 of these patients at less than six months of age, 8 between 2–3 years of age, 7 between 3–4 years of age, and 1 greater than 4 years of age. When asked why treatment was discontinued, the majority of clinicians reported that patients either already underwent a thyroid withdrawal challenge with normal thyroid testing off of medication or that this test was planned. One clinician reported parental non-compliance as the reason that treatment was discontinued.

Thyroid withdrawal challenge with decreasing/discontinuation of the dosage of levothyroxine with follow-up free T4/T4 and/or TSH was the re-evaluation testing of choice by the majority of respondents (81%). The timing of the thyroid challenge protocol varied (Table 3). Clinicians provided information about their protocol for initial thyroid testing following treatment cessation with 65% indicating they used specific protocol timing for re-testing thyroid function after treatment was held or discontinued. Of those that performed a thyroid withdrawal challenge, the majority (60%) indicated that they waited 4–8 weeks before rechecking thyroid labs.

## 3.3.2. Parent Responses

Asked how their child was diagnosed with CH, 94% of respondents identified the NBS heel prick method. Less than 4% of parents were unsure of the method of screening used to identify their child’s CH diagnosis. Of the 74 that indicated their child was started on therapy, 86% responded that their child was still on treatment. For those patients still on treatment, 35% responded that their doctor had temporarily discontinued the medication to do blood testing, 43% responded that this was not done and the doctor had informed them that it was a permanent problem, 5% responded that the doctor was planning to try to stop the medication in the future to confirm the permanency of the condition, and 17% were unsure of their doctor’s care plan. All parents who reported that their child’s treatment was discontinued also reported that the pediatric endocrinologist had stopped the treatment. Among those who responded, no parent indicated that they had stopped the medication on their own, and no parent reported social determinants (e.g., medication cost) as interfering with adherence.

### 3.4. Education and Genetic Counseling

#### 3.4.1. Clinician Responses

Clinicians were asked about the CH educational resources and genetic counseling services that their practice provides. Of the 214 clinicians who returned surveys, 182 provided information about CH education practices, and 160 provided information about genetic counseling (Table 4). A total of 74 clinicians reported that they provided some variety of both CH education and genetic counseling. The majority of clinicians (93%) provided face-to-face education, with over half providing printed literature. In terms of genetic counseling, 41% reported that their practice provided that service face-to-face and 45% reported that they provided no standard form of genetic counseling.

#### 3.4.2. Parent Responses

The majority of parents (99%) reported receiving some form of information from their child’s pediatric endocrinologist and/or their regular doctor at time of diagnosis (Table 5). Nearly half (49%) reported receiving printed materials on CH and utilizing the internet (42%) to search for information on their own in addition to receiving information from their child’s doctor. One parent reported receiving no education or information.

Finally, parents were asked if they were satisfied (Yes/No) with the information received about their child’s CH diagnosis. Of the parental responses, 65% were satisfied with the information they received. To test the relationship between parent satisfaction with information received and source of education, we used a chi-square test of independence. Sources of education that were significant (*p* < 0.05) for parents were pediatricians talking to the parent and receiving printed materials on CH. Of the parents who were dissatisfied (*n* = 26), 20 would have preferred more information from their child’s doctor, 17 wanted more from the state health department, and only 6 wanted more via the internet.

## 4. Discussion

In 2007, 409 children in our region were confirmed to have CH, indicating a birth prevalence of 1:1836 (range, 1:1472–2438). As noted in our previous report outlining the various logistical, regulatory, and legal challenges encountered during this study preparation and execution, we identified a number of methodologies and variables potentially influencing the state differences in birth prevalence [7]. These included state demographic differences, testing methods, and state-specific cutoff values, along with influences on diagnostic sensitivity, such as sample timing, gestational age, weight of the infant, use of certain therapies, such as blood products, and infant co-morbidities. In addition, infants with only transient abnormalities with their thyroid function studies were likely included, but evaluating specific details separating transient versus permanent primary CH was beyond the scope of this study. Transient primary CH may be defined as transient elevations in TSH which normalize at a later date and/or off thyroid replacement therapy.

This study is limited by its design and use of mailed, paper clinician and provider surveys. This includes data from infants in which no response was obtained as well as our inability to obtain data from Ohio prior to the end of the study. Even among those clinicians and parents who responded, some returned surveys were incomplete or had questions that were answered incorrectly invalidating certain data. Thus, we only had data on 214 of the 409 children (52.3%) diagnosed with CH in 2007. Despite these limitations, we demonstrated a clinician survey response rate of 64% and a parent rate of 26% of the surveys sent. This is acceptable when considering that most states only maintain parent addresses from the time of delivery.

Of the clinician respondents reporting on children that they were actively following, nearly all used laboratory testing after the initial NBS to confirm the diagnosis. However, only 53% had any form of imaging performed and the definitive etiology was reported as having been established in only 48% of infants. While there remains some controversy over the use of imaging at diagnosis, the benefits to establishing etiology are clearly defined in consensus guidelines from both the AAP and the ESPE [3,4]. The lack of definitive diagnosis, though, has implications when assessing the management history and procedures for re-evaluating whether the child requires lifelong levothyroxine therapy for permanent hypothyroidism.

Clinician respondents reported that 85% of patients were still managed with levothyroxine, but, of these, only 25% intended to re-evaluate the thyroid axis. Initial presentation and rise in TSH levels during infancy were the two most common reasons provided for this decision. Previous reports have raised concerns over parental non-compliance, including discontinuation of thyroid medication without clinician supervision, as a significant issue in children managed for CH [6]. In our sample, we had one clinician report parental non-compliance and three children were lost to follow-up. Furthermore, we had one parent report that thyroid treatment was stopped because the prescription ran out, but no clinician data was returned for that child.

In addition to inconsistent use of imaging modalities, the use and timing of an established protocol for re-evaluation, namely a “thyroid challenge”, also varied. Again, a thyroid challenge involves assessing thyroid function with laboratory studies after reducing or discontinuing thyroid medication. Even though the majority did have a protocol for a thyroid challenge, the timeframe for re-evaluation of thyroid function tests varied significantly. From those providing details of their protocol, 89% obtained thyroid function studies within 8 weeks after discontinuing levothyroxine. The AAP consensus guidelines suggest discontinuing for 30 days followed by retesting at the end of that 30 day period. The ESPE consensus guidelines suggest a graded dose reduction in increments of 30% with assessment to determine if TSH rises to equal or greater than 10 mU/L over 4–6 weeks [3,4]. Table 6 summarizes the recommendations for when to re-evaluate from the most recent consensus committee statement from the ESPE [4].

Beyond clinical management, family education is a key component to enhancing the care of a patient with CH. Family education consists of face-to-face discussion, as well as paper and electronic resources provided to the family. Historically, the need for genetic counseling has been quite limited as CH was identified as nearly an entirely sporadic disease (85–90% of cases) without identifiable genetic causes. As we detailed in our first CH study report, however, recent research demonstrates that there are a growing number of distinct genetic forms of isolated or syndromic thyroid dysgenesis and dyshormonogenesis [8]. Given the high percentage of clinicians reporting that definitive etiology was not demonstrated in their patients, our study results suggest that a consistent approach to education is needed. This includes medical education highlighting the extreme importance of levothyroxine therapy, lab monitoring, and clinical follow-up, as well as general genetic counseling to review dysgenesis and dyshormonogenesis, the two major forms of CH. Patients with a history suggestive of familial disease or phenotypic features raising the question of a syndromic disorder should receive directed genetic counseling as well as appropriate molecular genetic assessment.

Of our parent respondents in our study, only two-thirds were satisfied with the level of medical education and genetic counseling they received. While establishing a link between education and quality of care and follow-up was not possible using our survey design, our findings suggest that improved education and counseling was desired and could have positively influenced care. Based on our review, several studies have specifically examined general newborn screening educational materials available in the United States. These reports found materials lacking greatly in consistency, content, and language readability [9,10]. While public health education fact sheets for CH and levothyroxine therapy have been jointly developed at the national level by the AAP (Section of Endocrinology) and the Pediatric Endocrine Society, these provide only general information for families and have not been formally adopted by NBS programs [11]. With this in mind, we would recommend a systematic approach to medical education and counseling that allows for the differences in sociodemographic and educational backgrounds. Ideally, this would include formalized medical and genetic educational materials developed in collaboration with parents of children with CH, to be ratified and adopted within each state.

## 5. Conclusions

To our knowledge, this is the first multi-state study assessing NBS long-term follow-up factors related to clinical management and education practices of CH. Our results highlight a disparity in diagnostic birth prevalence, management and monitoring practices, and family education and genetic counseling. We believe this is in large part due to the varying levels of resources of individual clinicians, state public health departments, and state NBS programs. Our results support the need for the development of consistent best practice and educational guidelines, as well as readily available resources, adopted at the regional and national level.

## Figures and Tables

**Table 1:** Newborn screening and CH birth prevalence in Region 4 for 2007.

State	First-Tier Screening Method	Live Births	CH Diagnosis	Birth Prevalence
Michigan	TSH	125,172	85	1:1473
Minnesota	TSH	73,675	46	1:1602
Wisconsin	TSH	72,757	43	1:1692
Illinois	TSH	180,530	94	1:1920
Ohio	TSH	150,784	75	1:2010
Indiana	TSH	89,719	42	1:2136
Kentucky	T4/TSH	58,507	24	1:2438
Total	-	751,144	409	1:1836

CH—congenital hypothyroidism, TSH—thyroid-stimulating hormone, T4—thyroxine. Adapted from Wintergerst et al. J Genet Couns. 2015 Jun; 24(3):464–72.

**Table 2:** Clinicians report of studies used to confirm CH diagnosis *.

Laboratory Studies	*n*	%	Imaging Studies	*N*	%
TSH	162	97	Thyroid ultrasound	40	24
Free thyroxine (T4)	139	84	Thyroid technetium scan	50	29
Total T4	28	16	Thyroid uptake scan (I^123^)	3	2
Free triiodothyronine (T3)	8	5	Bone age X-ray	4	2
Total T3	5	3	Brain MRI/CT	1	<1
T3 Uptake	1	<1			
Thyroglobulin level	4	2			
TBII/TSH receptor antibody	4	3			
Anti-thyroid peroxidase and/or Anti-thyroglobulin antibodies	3	2			
Maternal thyroid testing	0	--			

* Respondents could choose all studies that applied. CH—congenital hypothyroidism, TSH—thyroid-stimulating hormone, TBII—thyrotropin-binding inhibitory immunoglobulin, MRI-magnetic resonance imaging, CT-computerized tomography.

**Table 3:** Clinicians report of protocol timing for re-evaluation of thyroid function following treatment cessation (thyroid challenge).

	*n* = 138	%
Within 3 months	134	97
2–4 weeks after discontinuation	40	29
4–8 weeks after discontinuation	83	60
8–12 weeks after discontinuation	11	8
Greater than 3 months	4	3
3–6 months after discontinuation	4	3
6–12 months after discontinuation	-	

**Table 4:** Percentage of clinicians reporting forms of CH education and genetic counseling provided by their practice.

Source	CH Education * (*n* = 182)	Genetic Counseling * (*n* = 160)
	*n* (valid %)	*n* (valid %)
Face-to-face education in office	170 (93)	65 (41)
Printed literature	105 (58)	40 (25)
Internet references	22 (12)	3 (2)
Information sent by state agencies	18 (10)	1 (<1)
No standard form of education provided	6 (3)	72 (45)
Referral to pediatric endocrinologist	3 (2)	3 (2)
Referral to genetic specialist for counseling	-	14 (9)

CH, congenital hypothyroidism, * Respondents could choose all that apply.

**Table 5:** Sources of education for parents satisfied with the information that they received compared to parents that would like to have received more information about their child’s CH diagnosis.

Source	Satisfied ^a^ (*n* = 48)	Not Satisfied ^a^ (*n* = 26)	χ^2^
	*n* (valid %)	*n* (valid %)	
Pediatrician talked to parent	30 (63)	9 (35)	5.26 *
Pediatric endocrinologist talked to parent	42 (88)	22 (85)	0.12
Genetic doctor/counselor talked to parent	2 (4)	1 (4)	<0.01
Printed materials on CH	29 (60)	7 (27)	7.57 **
Internet sites were referred to me	8 (17)	4 (15)	0.02
Information received in the mail	8 (17)	2 (8)	1.16
I looked up information on my own via the Internet	20 (42)	11 (42)	<0.01
No one explained the diagnosis or gave me information		1 (1)	

CH, congenital hypothyroidism, ^a^ Respondents could choose all that applied, * *p* < 0.05, ** *p* < 0.01

**Table 6:** Recommendations for re-evaluating the thyroid axis [4].

When to Re-Evaluate	When Re-Evaluation Can Be Waived
Definitive etiology not determined by diagnostic testing	Thyroid dysgenesis demonstrated by imaging (with exception of DUOX2 mutations or Pendred syndrome)
Initial treatment started for pre-term infants or during illness	Dyshormonogenesis confirmed by molecular genetic testing
Evidence of positive thyroid autoantibodies at diagnosis	TSH elevations above normal reference range for age after first year of life
No dosage increase in L-T4 needed since infancy as indicated by rise in TSH above normal range for age	
Negative molecular investigation for enzyme defect or whom no testing has been performed	
